# Polyethylene Glycol Versus Senna for Bowel Preparation for Colonoscopy in Children: Updated Evidence by a Systematic Review and Meta-Analysis

**DOI:** 10.7759/cureus.17813

**Published:** 2021-09-08

**Authors:** Jun Watanabe, Kazuhiko Kotani

**Affiliations:** 1 Community and Family Medicine, Jichi Medical University, Shimotsuke, JPN

**Keywords:** child nursing, colon disease, polyethylene glycol, senna, pediatric practice

## Abstract

For colonoscopy, bowel preparation, especially that using polyethylene glycol (PEG) or senna, is performed among children with gastrointestinal disorders; however, it is not fully grounded in evidence. This study reviewed via meta-analyses the approaches to bowel preparation for colonoscopy in children.

Electronic databases and trial registries were searched until April 2021. Quality assessment was conducted using the Grading of Recommendations, Assessment, Development, and Evaluation method.

In total, three randomized controlled trials (318 patients) were identified. PEG was observed as a preferred protocol of bowel preparation compared with senna (risk ratio [RR] 1.35, 95% confidence interval [CI] 1.05-1.74; I^2^ = 15%). It was less painful than senna (RR 0.62, 95% CI 0.44-0.87; I^2^ = 0%). No serious adverse events were noted. Overall, the certainty of the evidence was low to moderate.

PEG might be a preferred preparation agent for colonoscopy in children. Given the limited data, more studies are recommended.

## Introduction and background

Gastrointestinal disorders are commonly seen in children, and colonoscopy is considered the gold standard for diagnosis and treatment of patients with gastrointestinal disorders [1]. For example, colonoscopy is performed in children with inflammatory bowel disease, bleeding colitis, colon polyps, and malignancies [2-5]. Inadequate bowel preparation is known to adversely affect the colonoscopy procedure [6,7]. Bowel preparation is reported to be inadequate in one-third of colonoscopies and in approximately 5% of cases, and inadequate preparation led to cancellation or interruption of the examination [8]. The cost of cancelled or interrupted examinations has increased from 12% to 22% [9,10]. Even if bowel preparation is performed with consideration of the patient’s age, body size, and medical condition [1,11], a standard protocol for bowel preparation for children with gastrointestinal disorders is required.

In the clinical setting, osmotic drugs (i.e., polyethylene glycol) and stimulant laxatives (i.e., senna) are empirically used. Regarding the standard protocol for bowel preparation, an earlier systematic review [12] with three randomized controlled trials (RCTs) [13-15] reported that polyethylene glycol and senna demonstrated similar efficacies for bowel preparation before colonoscopy in children. However, that review [12] included one study [13] in which magnesium citrate, not senna, was compared to polyethylene glycol. After that review [12], new findings from an RCT have also been reported [16]. Thus, in order to update our knowledge concerning the use of polyethylene glycol and senna for colonoscopy, we reviewed via a meta-analysis the efficacy of polyethylene glycol compared to senna for bowel preparation before colonoscopy in children.

## Review

Study selection and outcomes

This study followed the preferred reporting items for systematic review and meta-analysis 2020 (PRISMA-2020) [17]. Inclusion criteria were RCTs that compared the use of polyethylene glycol versus senna in bowel preparation for colonoscopy in children with gastrointestinal disorders. The dosage of polyethylene glycol and senna were 1-3 g/kg/day and 1-3 mg/kg/day during 1-3 days, respectively. No restrictions to language, country, observation period, or publication year were applied. Exclusion criteria were studies on patients above 18 years or using non-colonoscopic procedures, such as flexible sigmoidoscopy, computed tomography-colonography, and capsule endoscopy. The primary outcomes were quality of bowel preparation and the number of patients with abdominal pain. The secondary outcome were all adverse events. The definition of adverse events was based on the guidelines [1,6,7]. Serious adverse events were defined as those requiring additional medication [1,6,7]. Preferred bowel preparation was defined as the number of patients with a score of excellent or good as assessed by the endoscopist, with at least 90% of the mucosa visible [18], corresponding to an excellent level on the Ottawa bowel preparation scale [19] and an excellent or good level on the Aronchick scale [20].

The following databases were searched: MEDLINE via PubMed, the Cochrane Central Register of Controlled Trials via Cochrane Library, Embase via Dialog, the World Health Organization International Clinical Trials Platform Search Portal (ICTRP) and ClinicalTrials.gov (Appendix 1). The reference lists of studies were checked with the international guidelines [1,6,7]. The studies were included in the lists if eligible RCTs cited the studies necessary for this review.

Data collection, the assessment of the risk of bias, and the analyses

Titles and abstracts were screened by independent reviewers, followed by an assessment of the eligibility based on the full text. Disagreements between reviewers were resolved by discussion. Data extraction and the risk of bias in the included studies were independently evaluated using the Risk of Bias 2 [21]. Disagreements between reviewers were discussed.

The relative risk ratios (RRs) and 95% confidence intervals (CIs) were pooled for the preferred bowel preparation and abdominal pain. An intention-to-treat analysis for all dichotomous data was performed. A random-effects meta-analysis was performed using a freely available software program, Review Manager (RevMan 5.4.1). Adverse events were summarized based on the definition used in the original article.

The assessment of heterogeneity, reporting bias, and certainty of evidence

The statistical heterogeneity was assessed by the visual inspection of the forest plots and analyzing the I^2^ statistic (I^2^ values of 0%-40%: may not be important; 30%-60%: potentially moderate heterogeneity; 50%-90%: potentially substantial heterogeneity; 75%-100%: considerable heterogeneity) [22]. In cases of substantial heterogeneity (I^2^ > 50%), the reason for the heterogeneity was evaluated by a subgroup analysis of the dosing periods for polyethylene glycol (1 versus 2 days). A sensitivity analysis for excluding studies with a high risk of bias was performed to assess whether or not the results of the present review were robust. The correct analysis of the earlier review [12] for excluding studies with the wrong intervention [13] was performed to assess the consistency with the results in the present review.

Clinical trial registry systems (ClinicalTrials.gov and ICTRP) were searched, and an extensive literature search was conducted for unpublished trials. We did not perform funnel plots or the Egger test because there were fewer than 10 trials according to the Cochrane handbook [22]. The certainty of evidence was independently assessed based on the Grading of Recommendations Assessment, Development and Evaluation (GRADE) approach [23]. A summary table of each study was made for the outcomes based on the Cochrane handbook [22].

Results

**Figure 1:**
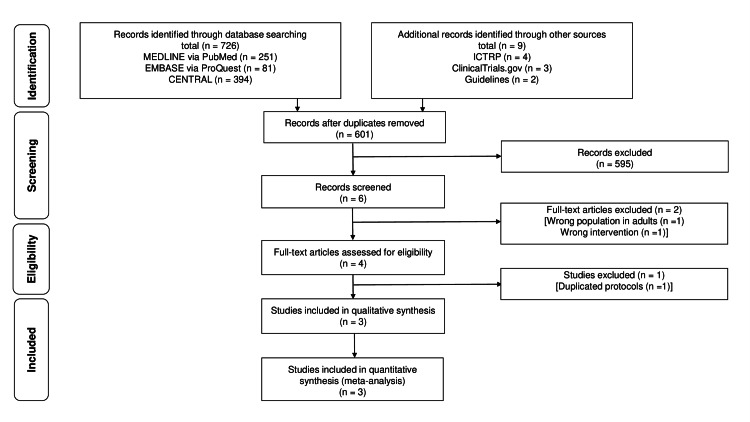
Flow chart of the article search

Figure 1 shows the flow chart of the article search. A total of 601 records were searched on April 21, 2021. After the initial screening of the title and abstract, six records were identified. After the full-text screening, two studies were excluded because the study was conducted on adult patients [24] or the wrong intervention was performed [13]. Ultimately, three studies (318 participants) were identified [14-16]. Although the documents and the references of the initially included studies were searched, no additional studies that met the inclusion criteria were identified.

Table 1 summarizes the characteristics of the eligible studies. Two trials used polyethylene glycol for two days [14,15], while the other used it for one day [16]. Bowel preparation was evaluated by numeric rating scale in one study [15], the Aronchick scale in one study [14], Ottawa bowel preparation scale in two studies [14,16], and Boston bowel preparation scale in one study [16]. Table 2 and Table 3 show the risk of bias. Overall, the risk of bias in all studies was of some concern because the randomization process was not clearly described, and the study protocols were not fully registered.

**Table 1:** Summary of the characteristics of the eligibility studies

Authors [ref no.]	Year	Country	Subject no. (polyethylene glycol/senna)	Age (years) (polyethylene glycol/senna)	Dosing periods of polyethylene glycol (days)	Dosing periods of senna (days)
Kierkus et al. [14]	2013	Poland	30 (16/14)	14.1/14.2	2	2
Terry et al. [15]	2013	USA	160 (80/80)	12.3/13.3	2	2
Tutar et al. [16]	2019	Turkey	128 (64/64)	10.3/10.1	1	3

**Table 2:** Quality scores for the eligibility studies for preferred bowel preparation

Authors [ref no.]	Risk of Bias 2 tool assessment					
	Bias arising from the randomization process	Bias due to deviations from intended interventions	Bias due to missing outcome data	Bias in measurement of the outcome	Bias in selection of the reported results	Overall risk of bias
Kierkus et al. [14]	Low	Low	Low	Low	Some concerns	Some concerns
Terry et al. [15]	Some concerns	Low	Low	Low	Low	Some concerns
Tutar et al. [16]	Some concerns	Some concerns	Some concerns	Low	Some concerns	Some concerns

**Table 3:** Quality scores of the eligibility studies for patients’ abdominal pain and adverse events

Authors [ref no.]	Risk of Bias 2 tool assessment					
	Bias arising from the randomization process	Bias due to deviations from intended interventions	Bias due to missing outcome data	Bias in measurement of the outcome	Bias in selection of the reported results	Overall risk of bias
Kierkus et al. [14]	Low	Low	Low	Low	Some concerns	Some concerns
Terry et al. [15]	Some concerns	Low	Low	Low	High risk	High risk
Tutar et al. [16]	Some concerns	Some concerns	Some concerns	Low	Some concerns	Some concerns

Outcomes

**Figure 2:**
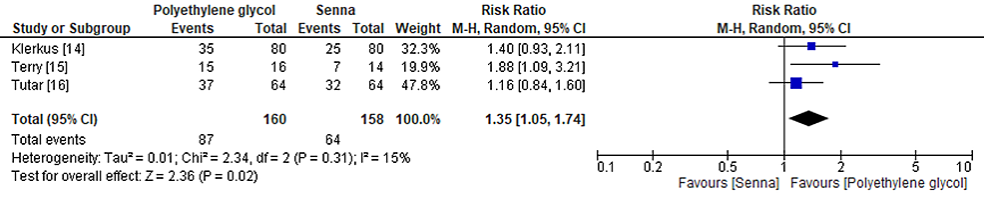
Forest plot of preferred bowel preparation CI, confidence interval; M-H, Mantel-Haenszel.

**Figure 3:**
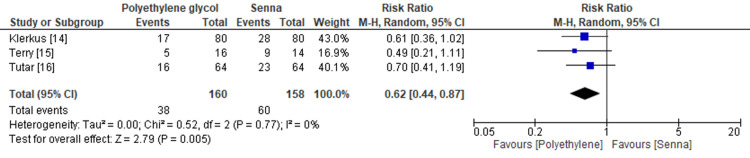
Forest plot of abdominal pain CI, confidence interval; M-H, Mantel-Haenszel.

Table 4 shows the summary of findings using the GRADE approach [23]. Polyethylene glycol was found to be the preferred protocol of bowel preparation compared with senna (RR 1.35, 95% CI 1.05-1.74; I^2^ = 15%) (Figure 2). The certainty of evidence was moderate because of imprecision due to the small sample size. Polyethylene glycol was also shown to be less painful than senna (RR 0.62, 95% CI 0.44-0.87; I^2^ = 0%) (Figure 3). The certainty of evidence was low because of imprecision due to the small sample size and high risk of bias.

**Table 4:** Summary of findings CI, confidence interval; RR, risk ratio; GRADE, Grading of Recommendations Assessment, Development and Evaluation. *The risk in the intervention group (and its 95% CI) is based on the assumed risk in the comparison group and the relative effect of the intervention (and its 95% CI). ^a^Downgraded because of imprecision due to the small sample size. ^b^Downgraded because of imprecision due to high risk of bias.

Polyethylene glycol versus senna for bowel preparation for colonoscopy in children						
Patient or population: children, Setting: colonoscopy, Intervention: polyethylene glycol, Comparison: senna						
Outcomes	Anticipated absolute effects* (95% CI)		Relative effect (95% CI)	Patient number (studies)	Certainty of the evidence (GRADE)	Comments
	Risk with senna	Risk with polyethylene glycol				
Quality of bowel preparation	405 per 1000	547 per 1000 (425-705)	RR 1.35 (1.05-1.74)	318 (3 RCTs)	Moderate^a^	Polyethylene glycol was observed as a preferred protocol of bowel preparation
Abdominal pain	380 per 1000	235 per 1000 (167-330)	RR 0.62 (0.44-0.87)	318 (3 RCTs)	Low^a,b^	Polyethylene glycol did not cause abdominal pain
Adverse events	In three studies, both groups had similar untreated adverse events, such as nausea, vomiting, and sleep disorders between both groups			318 (3 RCTs)	Low^a,b^	No serious adverse events were observed

**Figure 4:**
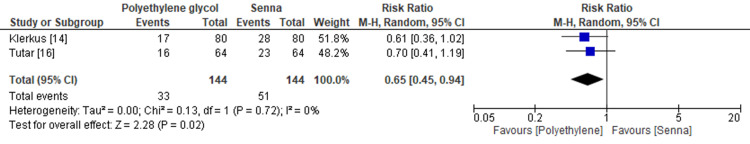
Prespecified sensitivity analyses CI, confidence interval; M-H, Mantel-Haenszel.

**Figure 5:**
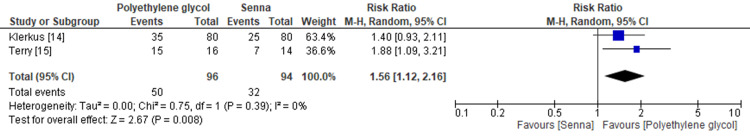
Correct analysis of preferred bowel preparation in excluding studies with a high risk of bias CI, confidence interval; M-H, Mantel-Haenszel.

**Figure 6:**
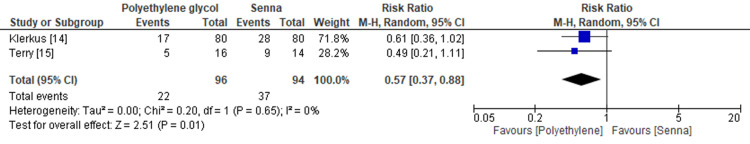
Correct analysis of abdominal pain in excluding studies with a high risk of bias CI, confidence interval; M-H, Mantel-Haenszel.

In all three studies [14-16], the prevalence of adverse events such as nausea, vomiting, and sleep disturbances was similar between polyethylene glycol and senna. No serious adverse events requiring additional medication were observed. In addition, a subgroup analysis was not performed due to the lack of substantial heterogeneity (I^2^ > 50%). The prespecified sensitivity analyses were consistent with the primary findings of the present review (Figure 4). Furthermore, the correct analysis of the earlier review was also consistent with the primary findings of the present review (Figure 5 and Figure 6).

Discussion

The present systematic review and meta-analysis suggested that polyethylene glycol might be a preferred preparation for colonoscopy in children with gastrointestinal disorders. As the evidence was moderate to low, the results should be interpreted with caution in the clinical setting. However, the updated findings of integrated RCTs on polyethylene glycol will further facilitate the establishment of a standard protocol for bowel preparation in children.

The earlier systematic review [12] reported a similar efficacy for preparing the bowel for colonoscopy between polyethylene glycol and senna (RR 0.73, 95% CI 0.31-1.76; I^2^ = 95%) according to pooled data. Here, we reconsidered one study that did not compare polyethylene glycol and senna [13] in that review [12] and further added a recent study that favored polyethylene glycol over senna [16]. Thus, the results of this updated systematic review differed from the previous review.

In bowel preparation of polyethylene glycol and senna, abdominal-related symptoms such as abdominal pain and the others (e.g., nausea, vomiting) can adversely appear [14-16]. The present review showed that such events were mildly observed in some cases, and none required nasogastric tube placement or hospitalization. These results were similarly reported to the previous review [12]. On the other hand, the present review found that senna leads to a higher incidence of abdominal pain than that of polyethylene glycol [14-16]. This may be explained by the fact that senna is a laxative that stimulates the movement of the intestinal tract and can induce abdominal pain [24]. Some previous studies on children also reported a high incidence of abdominal pain by senna [25-28]. Empirically, even though both polyethylene glycol and senna have been safely used for children and no serious adverse events were noted, we should be careful about abdominal pain as induced by senna.

There have been several bowel preparation quality scores. The most well-established and popularly used validated scores include the Aronchick scale [20], Boston bowel preparation scale [29], and Ottawa bowel preparation scale [19]. Although a previous review [29,30] described that the Boston bowel preparation scale should be used in clinical practice, all scales had the several limitations. Comparisons between these scales would be still needed for bowel preparation using polyethylene glycol in children.

The limitations associated with the present study warrant mention. First, as mentioned above, the sample size was not very large, although a rigorous methodology was adopted based on the PRISMA statement [17]. Second, the studies reviewed used different doses and dosing periods, although the heterogeneity for all studies was not shown. Third, the risk of bias in all studies reviewed was of some concern because the randomization approaches and protocols were not detailed. Further studies will be required to increase the certainty and generalizability of the evidence.

## Conclusions

We reviewed using a meta-analysis to assess the efficacy of polyethylene glycol and senna in bowel preparation before colonoscopy in children. Our review provided updated evidence suggesting that polyethylene glycol might be the preferred agent for use in bowel preparation before colonoscopy in children. The present study updated our knowledge on the use of polyethylene glycol and senna for colonoscopy. More studies, including RCTs, are needed to establish the efficacy of polyethylene glycol given the limited number of available studies for review.

## Appendices

Appendix 1. Search strategy

CENTRAL

#1. MeSH descriptor: [Colonoscopy] explode all trees

#2. colonoscop*:ti,ab,kw (Word variations have been searched)

#3. #1 OR #2

#4. MeSH descriptor: [Infant] explode all trees

#5. MeSH descriptor: [Child] explode all trees

#6. MeSH descriptor: [Adolescent] explode all trees

#7. (infant* OR child* OR pediatric* OR paediatric* OR adolescent* OR neonat* OR toddler OR young):ti,ab,kw (Word variations have been searched)

#8. #4 OR #5 OR #6 OR #7

#9. MeSH descriptor: [Cathartics] explode all trees

#10. MeSH descriptor: [Cathartics] explode all trees

#11. MeSH descriptor: [Polyethylene Glycols] explode all trees

#12. MeSH descriptor: [Sennosides] explode all trees

#13. (Cathartics OR laxative OR “colon lavage” OR “intestine preparation”):ti,ab,kw (Word variations have been searched)

#14. (PEG OR polyethylene OR macrogol OR movicol OR idrolax OR miralax OR transipeg OR forlax OR colyte OR golytely OR isocolan OR nulytely):ti,ab,kw (Word variations have been searched)

#15. (Senna OR Sennosides):ti,ab,kw (Word variations have been searched)

#16. # 9 OR #10 OR #11 OR #12 OR #13 OR #14 OR #15

#17. #3 AND #8 AND #16

MEDLINE

#1. Colonoscopy[Mesh]

#2. Colonoscop*[tiab]

#3. #1 OR #2

#4. Infant[Mesh]

#5. Child[Mesh]

#6. Adolescent[Mesh]

#7. (infant*[tiab] OR child*[tiab] OR pediatric*[tiab] OR paediatric*[tiab] OR adolescent*[tiab] OR neonat*[tiab] OR toddler[tiab] OR young[tiab])

#8. #4 OR #5 OR #6 OR #7 OR #8

#9. Cathartics[Mesh]

#10. “laxative”[Mesh]

#11. “Polyethylene Glycols”[Mesh]

#12. Sennosides[Mesh]

#13. Cathartics[tiab] OR laxative[tiab] OR “colon lavage”[tiab] OR “intestine preparation”[tiab]

#14. (PEG[tiab] OR polyethylene[tiab] OR macrogol[tiab] OR movicol[tiab] OR idrolax[tiab] OR miralax[tiab] OR transipeg[tiab] OR forlax[tiab] OR colyte[tiab] OR golytely[tiab] OR isocolan[tiab] OR nulytely[tiab])

#15. Senna[tiab] OR Sennosides[tiab]

#16. #9 OR #10 OR #11 OR #12 OR #13 OR #14 OR #15

#17. (randomized controlled trial [pt] OR controlled clinical trial [pt] OR randomized [tiab] OR drug therapy[sh] OR placebo [tiab] OR randomly[tiab] OR trial[tiab] OR groups[tiab]) NOT (animals [mh] NOT humans [mh])

#18. #3 AND #8 AND #16 AND #17

Embase

S1. EMB.EXACT.EXPLODE("colonoscopy")

S2. ti(colonoscopy) OR ab(colonoscopy)

S3. S1 OR S2

S4. EMB.EXACT.EXPLODE("child")

S5. EMB.EXACT.EXPLODE("adolescent")

S6. ab(infant OR child OR pediatric OR paediatric OR adolescent OR neonat OR toddler OR young) OR ti(infant OR child OR pediatric OR paediatric OR adolescent OR neonat OR toddler OR young)

S7. S4 OR S5 OR S6

S8. EMB.EXACT.EXPLODE("laxative")

S9. EMB.EXACT.EXPLODE("macrogol derivative")

S10. EMB.EXACT.EXPLODE("Senna")

S11. ab(PEG OR polyethylene OR macrogol OR movicol OR idrolax OR miralax OR transipeg OR forlax OR colyte OR golytely OR isocolan OR nulytely) OR ti(PEG OR polyethylene OR macrogol OR movicol OR idrolax OR miralax OR transipeg OR forlax OR colyte OR golytely OR isocolan OR nulytely)

S12. ab(Senna OR Sennosides) OR ti(Senna OR Sennosides)

S13. S8 OR S9 OR S10 OR S11 OR S12

S14. S3 AND S7 AND S13

S15. (ab(random*) OR ti(random*)) OR (ab(placebo*) OR ti(placebo*)) OR (ab(double NEAR/1 blind*) OR ti(double NEAR/1 blind*))

S16. S14 AND S15

ClinicalTrial.gov

Colonoscopy AND (PEG OR polyethylene OR macrogol OR movicol OR idrolax OR miralax OR transipeg OR forlax OR colyte OR golytely OR isocolan OR nulytely) AND (Senna OR Sennosides)

ICTRP

Colonoscopy AND (PEG OR polyethylene OR macrogol OR movicol OR idrolax OR miralax OR transipeg OR forlax OR colyte OR golytely OR isocolan OR nulytely) AND (Senna OR Sennosides)
